# Instrumented micro/macroindentation of porosity defects in laser powder bed fusion 316L stainless steel

**DOI:** 10.1007/s10853-025-10995-3

**Published:** 2025-06-05

**Authors:** Jordan S. Weaver, Houshang Yin, Jesse Redford, Xiaoyuan Lou

**Affiliations:** 1 Engineering Laboratory, National Institute of Standards and Technology, Gaithersburg, MD 20899 USA; 2 Department of Materials Engineering, Auburn University, Auburn, AL 36849 USA; 3 School of Materials Engineering, Purdue University, West Lafayette, IN 47907 USA; 4 School of Nuclear Engineering, Purdue University, West Lafayette, IN 47907 USA

## Abstract

Porosity defects and their effect on mechanical properties remain a concern in metals-based laser powder bed fusion, even for optimized process parameters. Instrumented micro/macroindentation with Berkovich and spherical tips to forces of 50 N and 150 N, respectively, were used to characterize three levels of porosity (fully dense, 1%, and 4%) created by decreasing and increasing the hatch spacing and two levels of heat treatments (stress-relieved and solution annealed). The spherical indentation data were converted to effective stress–strain curves along with an estimated modulus and yield stress. The Berkovich data were analyzed using the Oliver-Pharr method for modulus and hardness, and the hardness was used to estimate yield stress. The indentation results were compared against tensile results for the loading axis along the build direction (Z) and perpendicular to the build direction (X/Y). The modulus results largely agree for indentation and tensile tests. The average yield stress estimated from these indentation measurements captures the difference in heat treatment but does not capture subtle changes in porosity and test direction compared to tensile yield stress measurements. However, the indentation results show a considerable increase in scatter from test to test with the presence of porosity due to varying amount of porosity within the interaction volume under the indenter.

**Graphical Abstract:**

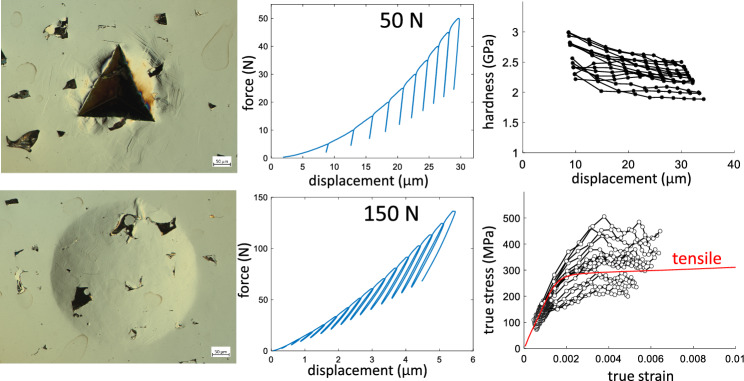

## Introduction

Laser powder bed fusion (L-PBF) is one of many additive manufacturing (AM) technologies that can produce complex, custom components with a short lead time compared to castings or subtractive manufacturing processes. While metals-based L-PBF can produce fully dense parts, porosity flaws remain a concern. A complete list of the mechanisms that create porosity in L-PBF can be found in several reviews [1–3]. The two most common categories of porosity flaws occur from a lack of sufficient energy (i.e., lack-of-fusion) or an overabundance of energy (i.e., unstable keyholing). This can occur within a single track (i.e., a single weld) or as the cumulative effect of multiple tracks or layers. The processing space for L-PBF is typically mapped out to avoid the regions where porosity flaws increase (e.g., [4]). However, a lack of sufficient energy and an abundance of energy leading to porosity flaws can still occur when operating with optimized process parameters due to shifts in the process by uncontrolled variables.

Uncontrolled L-PBF phenomenon include process by-products such as spatter and the vapor plume, which have been measured and quantified by various metrics correlated with lack-of-fusion porosity. Esmaeilizadeh et al. [5] observed a spatter rich region within the build area with large deposits leading to increased surface roughness and porosity. Snow et al. [6] found a statistically significant, causal relationship between spatter observed with in situ process monitoring measurements and porosity in the fabricated parts determined with X-ray computed tomography. Tenbrock et al. [7] studied a multi-laser L-PBF process finding a correlation between the plume propagation from one laser into the path of another laser and the occurrence of porosity defects. Another uncontrolled scenario leading to insufficient energy input and an increased probability for lack-of-fusion occurs if the optimal process parameters are operated in a colder condition (i.e., great heat flux away from the molten zone). Williams et al. [8] showed that lower surface temperatures on the component caused by longer inter-layer cooling times (i.e., the time between layers) and a short conduction path for material close to the build plate led to an increase in lack-of-fusion defects. Such conditions occur naturally because the printing process is typically operated with simple path planning rules without more complex strategies such as model-based control or real-time feedback control.

On the opposite end of the process window, unstable keyholing porosity can occur from uncontrolled conditions. Local overheating occurs when the laser path scans in a small area and when there is a short inter-layer dwell time. Baldi et al. [9] studied the effect of the inter-layer cooling time (i.e., the time between laser exposure of a given part from one layer to the next layer), which can vary considerably for multi-laser L-PBF machines. They found that a short inter-layer cooling time of 2 s had a higher average ± standard deviation porosity (0.96% ± 0.38%) compared to a longer time of 22 s (0.021% ± 0.16%). This also correlated with a higher melt pool depth for the 2 s inter-layer cooling time. Additionally, Baldi et al. [9] found that the porosity and melt pool depth increased for specimens with poorer heat flux or poorer heat dissipation. This was true for tall specimen geometries with cross-sectional areas that narrowed as they approached the build platform (porosity of 0.284% ± 0.128%) compared to a baseline condition (porosity of 0.029% ± 0.011%). Similarly, Rezaeifar and Elbestawi concluded that a porosity-free process zone could be maintained if the molten pool temperature was maintained in a safe range; otherwise porosity increased if the molten pool temperature was too hot or too cold [10]. Another high heat input scenario occurs at the start or end of laser tracks depending on the control method and timing of the laser power and galvanometer (galvo). Yeung et al. [11] demonstrated a reduction in porosity flaws when using a constant galvo speed at the end of the laser track (i.e., skywriting) compared to an exact stop method where the galvo decelerates with the laser still on. The slowdown of the galvo with full power delivery leads to an increase in the heat input and keyhole type porosity. These several examples illustrate that porosity flaws remain a concern in metals-based L-PBF due to shifts in the process toward cold or hot scenarios.

The main concern with porosity flaws in metals-based L-PBF is their potential detriment to mechanical properties. The two most commonly reported effects for small amounts of porosity are a reduction in tensile ductility and a reduction in fatigue life [2, 12, 13]. In general, porosity levels less than 1% have little effect on tensile properties [2, 12]. There are exceptions, and a reduction in tensile ductility is correlated with an increase in porosity even for small levels of porosity (e.g., [14, 15]). Porosity levels greater than a few percent, which is not typical of an optimized process, begin to reduce all tensile properties including Young’s modulus and yield stress [2, 16, 17]. The total porosity is only a first level indicator of tensile properties. In other words, the mechanical properties show different sensitivity to spherical pores and irregular lack-of-fusion pores where a greater reduction in tensile properties is observed for lack-of-fusion type porosity [17]. Lack-of-fusion porosity with a preferential pore orientation or preferential plane of defects can also create anisotropic elongation [18]. This study is specifically focused on tensile properties (modulus and yield stress) estimated from indentation measurements of L-PBF 316L stainless steel in the presence of porosity defects.

Indentation testing offers a means to probe the mechanical behavior at specific locations within a component. This is particularly desirable for additive manufacturing where the microstructure and mechanical properties within a single component may vary due to inherent process variation. Hardness and porosity show the same behavior as the tensile yield stress and ultimate strength where hardness decreases with increasing porosity for significant porosity levels [19, 20]. Multiple studies on L-PBF 316L stainless steel report Vickers hardness trends with porosity in the lack-of-fusion regime through the excessive energy input regime with an increase in spherical porosity [20–22]. Tucho et al. [20] report Vickers hardness dependence on the specimen orientation. A higher hardness occurred when indenting along the build direction compared to transverse to the build direction. This was the most pronounced for the lowest porosity specimens (0.14%) and was less pronounced for specimens with higher amounts of porosity (as high as 3.43%). Bakhtiarian et al. [21] report a linear trend of increasing Vickers hardness with increasing density for samples with a relative density of 96.3% to 99.5%.

In this study, L-PBF 316L stainless steel samples were printed with lack-of-fusion porosity, fully dense, and keyhole porosity by controlling the hatch spacing. Spherical tip (radius of 6.35 mm) and Berkovich tip instrumented indentation was employed in the micro- to macroindentation regime. This ensures an interaction zone under the indenter tip that incorporates porosity flaws. The indentation measurements were then used to estimate the tensile properties and stress–strain response using existing conversions in the literature.

## Materials and methods

### Material, structure, and tensile properties

The fabrication of specimens, microstructure characterization, and tensile testing have been reported by Kim et al. [23]. Important details are briefly described here. Specimens were manufactured using a commercial L-PBF machine (Concept Laser^1^ Mlab 100 R Cusing) with virgin, nitrogen atomized 316L stainless steel (SS) powder feedstock from Carpenter Powder Products that was sieved to 15–44 µm. The L-PBF machine is equipped with a Yb:YAG fiber laser (1070 nm wavelength) with a focus D4σ spot diameter of 50 μm and a maximum power of 100 W. An island scanning strategy was used with checkerboard patterns of 5 mm × 5 mm squares. The programmed laser power, scan speed, and solid layer thickness were kept constant at 90 W, 600 mm/s, and 25 μm, respectively. The hatch spacing (spacing between laser tracks) was decreased above the default to introduce unstable melting (i.e., keyhole) porosity and increased above the default to introduce lack-of-fusion porosity. The hatch spacing was 50 μm, 80 μm (default), and 150 μm. Two different heat treatments were applied: a stress-relief (SR) heat treatment at 650 °C for 1 h followed by air-cooling and a solution-anneal (SA) heat treatment at 1121 °C for 2 h followed by water quenching. The stress-relief heat treatment was applied to reduce residual stress while keeping as much of the as-built microstructure as possible, and the solution-anneal heat treatment was applied to create a more homogenous microstructure. These do not alter the porosity defects that were created during the printing process. This results in six different material states (three hatch spacings and two heat treatments).

The three different printing parameters were used to produce witness cubes (20 mm) and 20 mm × 20 mm × 12 mm samples were printed for indentation testing. Heat treatments were applied on whole samples prior to sectioning. Porosity was determined from X-ray computed tomography (XCT) [23]. The porosity levels for the 50 μm, 80 μm, and 150 μm hatch spacing were $\cong$ 1%, < 0.1%, and $\cong$ 4%. The 50 μm hatch spacing primarily had large irregular pores, whereas the 150 μm hatch spacing had a broader distribution of pore sizes that follow the scan strategy pattern due to insufficient melting between laser tracks. The grain morphology was determined from electron backscatter diffraction (EBSD) and optimal microscopy [23]. The SR material exhibited the typical L-PBF microstructure with elongated grains in the build direction. Features in the as-built microstructure such as the melt pool boundaries, dendritic cells, and dislocation cells are preserved in the SR material. The SA heat treatment eliminates these features and has larger, mostly recrystallized equiaxed grains.

Vertical tensile specimens were printed to net shape with a gauge length, thickness, and width of 25 mm, 2 mm, and 6 mm, respectively. Horizontal tensile specimens were printed and sliced from a thicker block to avoid the influence of support structures. All specimens were ground to the same surface finish. See Ref. [23] for more details. Tests with the tensile axis aligned with the build direction are referred to as “Z” and specimens with the tensile axis perpendicular to the build direction are referred to as “X/Y”. The average and standard deviations in Table 1 come from three tests per direction. There is some anisotropy where the X/Y specimens show higher strength with lower elongation compared to the Z specimens. The greatest difference in strength is between SR and SA heat treatments with a higher strength for SR specimens. The porosity introduced by off-nominal hatch spacing has the biggest impact on elongation followed by ultimate tensile strength (UTS) and yield stress.

**Table 1:** Data from Ref. [23]

Property	SR-50 μm	SR-80 μm	SR-150 μm	SA-50 μm	SA-80 μm	SA-150 μm
Z—E (GPa)	184.2 ± 5.5	186.9 ± 2.2	182.1 ± 6.4	187.4 ± 5.8	191.8 ± 2.6	182.6 ± 7.7
X/Y—E (GPa)	183.8 ± 2.2	184.6 ± 5.1	182.0 ± 4.6	188.7 ± 2.6	189.6 ± 6.5	177.5 ± 4.2
Z—YS (MPa)	452.1 ± 1.8	468.1 ± 3.5	466.9 ± 3.0	286.6 ± 2.9	292.2 ± 7.9	290.6 ± 2.1
X/Y—YS (MPa)	480.4 ± 3.5	528.0 ± 2.9	482.4 ± 2.4	337.1 ± 5.9	362.0 ± 3.5	326.2 ± 2.5
Z—UTS (MPa)	594.6 ± 3.2	602.9 ± 2.0	601.0 ± 2.2	563.3 ± 9.3	605.0 ± 9.0	584.3 ± 2.9
X/Y—UTS (MPa)	601.0 ± 0.8	689.2 ± 1.3	630.5 ± 3.3	603.7 ± 25.2	686.3 ± 9.0	669.8 ± 1.8
Z—EL (%)	51.4 ± 1.0	51.6 ± 0.8	37.3 ± 2.44	52.3 ± 1.6	60.0 ± 0.9	39.3 ± 0.4
X/Y—EL (%)	14.5 ± 1.0	45.7 ± 0.1	30.6 ± 0.8	21.3 ± 3.9	48.2 ± 2.0	39.0 ± 3.1

Average ± standard deviation tensile properties for Z and X/Y specimens. The tensile properties are Young’s modulus (E), yield stress (YS), ultimate tensile strength (UTS), and elongation at failure (EL)

Optical images of the residual indents were acquired at a total magnification of 200 × and pixel size of 0.174 μm using circular differential interference contrast (C-DIC) to qualitatively determine the residual indent size and deformation surrounding the indent relative to porosity defects. A coherent scanning interferometer (CSI) with a 1024 pixel × 1024 pixel array was used to measure the topography of two representative residual indents to quantify the differences in pile-up between the Berkovich and spherical indents. The Berkovich indent height map was collected with a 50 × Mirau objective with 0.5 × tube lens, numerical aperture of 0.55, 340× 340 μm field of view, an optical resolution of 0.52 μm using Sparrow criterion, and spatial sampling 0.327 μm. The spherical indent height map was collected with a 20 × Mirau objective with 1 × tube lens, numerical aperture of 0.4, 420 × 420 μm field of view, optical resolution of 0.71 μm, and spatial sampling of 0.409 μm.

### Indentation

#### Test parameters

316L SS specimens for indentation were 20 mm × 20 mm × $\cong$ 10 mm (approximately 2 mm was removed during removal from the build platform and sample preparation). All specimens were mounted in resin and remained mounted during indentation testing. The top surface was ground, and mirror polished using 1 μm diamond suspension followed by colloidal silica. While still mounted, the bottom surface was ground to expose the base of the metal sample and glued with a thin layer of glue (Loctite liquid super glue) to a hardened steel plate. This was done to reduce compliance while securing samples to fixed positions. Indentation along the Z-direction was performed first. After this, the samples were broken out of the mounts and then cross-sectioned into two 20 mm × $\cong$ 10 mm × $\cong$ 10 mm pieces for indents in the X/Y-direction. The same mounting and polishing procedures were used for X/Y samples. All indentation testing was performed using a screw-driven load frame for instrumented indenter (Zwick ZHU 2.5) with a 2.5 kN load cell (Class 1 calibration) and an extensometer with a displacement measurement resolution of 2 nm (Class 0.2 calibration). The machine compliance was determined by the manufacturer in accordance with ISO 14577 using a best fit line intercept to the reciprocal of the unloading stiffness to 1 over the square root of the force [24]. A tungsten carbide spherical indenter with a 6.35 mm radius was used for spherical indentation. Indirect calibrations for Rockwell hardness were carried out on HRR scale and HR15 scale calibration blocks periodically as a quality check of the machine and tip. In addition to the spherical tip, a diamond Berkovich tip was used, which had an average radius of 350 nm according to the manufacturer.

**Figure 1:**
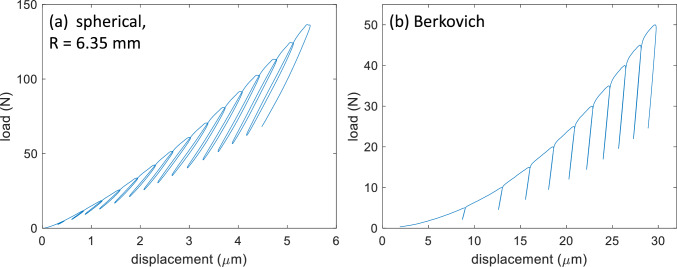
Representative load–displacement curves (**a**) spherical tip (**b**) Berkovich tip.

The test program for both tips involved loading and partial unloading (50% of the maximum force) in cycles with an incremental increase in depth or force after each load-unload cycle. Example force–displacement curves are shown in Fig. 1. The test programs were tailored to each tip based on the machine operation and the desired number of points in the final analysis. For spherical indents, the first unload occurred at 5 N followed by an increase in depth of 0.4 μm per cycle for a total of 15 cycles. A first unload at 5 N was selected so that a continuous elastic loading segment would occur without obstruction by a load-unload cycle and without causing too much plastic strain past the initial elastic deformation. The depth increment was selected to provide a reasonable spacing of indentation stress–strain points on the resultant indentation stress–strain curve. Note that using a load increment tends to crowd these points on the indentation stress–strain curve as the contact area increases due to the nature of a spherical tip. Loading and unloading occurred at rates of 0.5 mm/min and 10 N/s, respectively. The choice of displacement control during loading was selected because the target increment is a displacement, and the choice of load control during unloading was selected because the target is a force (50% of maximum force). This choice and the rates were primarily selected to produce data with less noise and overshoot at the switch between loading and unloading and vice versa while balancing the overall time for each test. For Berkovich indents, the first unload also occurred at 5 N with an increase in force of 5 N per cycle for a total of 10 cycles. A first unload at 5 N was selected so that the contact displacement was large enough to satisfy the requirements for using an ideal area function. A force target for the first unload also produced more consistent curves because the target is less sensitive to the zero-point/first contact determination. The load increment provides sufficient spacing of data points on modulus and hardness versus depth curves. Loading and unloading occurred at rates of 10 N/s and 0.1 mm/min, respectively. Similarly, the parameters were primarily selected to produce data with less noise and overshoot at the switch between loading and unloading and vice versa while balancing the overall time for each test. This includes the choice of displacement control for the unloading in Berkovich tests. The slowest reasonable load control tended to be 10 N/s while slower and smoother machine operation was achieved with displacement control at 0.1 mm/min. For both tips, unloading is assumed to be primarily elastic, and this step is necessary to calculate the contact area since it evolves during the test. Each unload results in a modulus and hardness measurement for the Berkovich tip and an indentation stress and strain data point for the spherical tip. The incremented load-unload cycles allow for observations of how these terms change with depth or strain at each test location. Note that the maximum forces and displacements are very different for the two tips and test programs because the tip geometries are very different. Hence, the programmed loading–unloading parameters are also different. The spherical indents are meant to capture the initial elastic-to-plastic transition, whereas the sharp nature of the Berkovich tip imposes a constant and nearly immediate amount of plastic deformation. Residual indents were on the order of 300 µm to 400 µm across. Indent arrays typically included 16 tests with 1.3 mm center-to-center spacing between indents.

#### Indentation data analysis

The spherical load–displacement ($P, h$) data were normalized to an effective stress–strain (${\sigma }_{ind}, {\varepsilon }_{ind}$) response, Eqs. (1) and (2), following the analysis of Kalidindi and Pathak that was first applied to spherical nanoindentation [25]. This was later extended to spherical macroindentation [26]. Here the term ${h}_{s}$ refers to the sample displacement, which accounts for a small elastic deformation of the tip according to Ref. [26]. The normalization of the load–displacement data uses Hertz’s theory, Eqs. (3) and (4), to determine the effective modulus,${E}_{eff}$, and a displacement correction (see Ref. [26]) from the initial elastic loading, and the contact radius,$a$, from the unloading stiffness,$S$. The unloading stiffness,$S=dP/dh$, is determined from a regression of the unloading force–displacement data [26]. The effective modulus is a combination of the sample ($s$) and indenter ($i$) Young’s modulus,$E$, and Poisson’s ratio,$v$, Eq. (5), and the effective radius,${R}_{eff}$, is a combination of the sample and indenter radius of curvature, Eq. (6). For the tungsten carbide tip, 540 GPa and 0.2 were used for the modulus and Poisson’s ratio, respectively, and a sample Poisson’s ratio of 0.3 was assumed for stainless steel. During the initial elastic loading, ${R}_{s}=0$ for the flat surface, and${R}_{eff} = {R}_{i}$. Additional details of the analysis can be found in Ref. [26].

1 $${\sigma }_{ind}=\frac{P }{\pi {a}^{2}}$$

2 $${\varepsilon }_{ind}=\frac{3\pi }{4}\frac{{h}_{s}}{a}$$

3 $$P=\frac{4}{3}{E}_{eff}{R}_{eff}^\frac{1}{2}{h}_{e}^\frac{3}{2}$$

4 $$a=\frac{S}{{2E}_{eff}}$$

5 $$\frac{1}{{E}_{eff}}=\frac{1-{v}_{s}^{2}}{{E}_{s}}+\frac{1-{v}_{i}^{2}}{{E}_{i}}$$

6 $$\frac{1}{{R}_{eff}}=\frac{1}{{R}_{s}}+\frac{1}{{R}_{i}}$$

The Berkovich load–displacement ($$P, h)$$ data were analyzed using the Oliver-Pharr method to determine the sample Young’s modulus and hardness [27], Eqs. (7) and (8). The effective modulus is related to the indenter and sample elastic properties using the same Eq. (5). For the diamond tip, 1140 GPa and 0.17 were used for Young’s modulus and Poisson’s ratio, respectively. The contact area, $$A$$, was determined with the ideal geometry assumption, Eq. (9). This is acceptable so long as the contact depth, $${h}_{c}$$, for the shallowest measurement is > 6 μm according to ISO 14577–1 [28]. The unloading stiffness, $$S$$, was determined using Eq. (10) where $$m, B,$$ and $$hp$$ are determined by a regression fit of the unloading data to the power law relationship of $$P=B{\left(h-{h}_{p}\right)}^{m}$$. The contact depth, $${h}_{c}$$, is determined using Eq. (11). The factor $$\varepsilon$$ was calculated using the approximation formula from Ref. [29] according to ISO 14577–1. It should be noted that ISO 14577 does not provide guidance on the use for porous materials.

7 $$H=\frac{P}{A}$$

8 $${E}_{eff}=\frac{\sqrt{\pi }}{2} \frac{S}{\sqrt{A}}$$

9 $$A=24.49{h}_{c}^{2}$$

10 $$S=mB{\left(h-{h}_{p}\right)}^{m-1}$$

11 $${h}_{c}=h-\frac{\varepsilon P}{S}$$

The indentation measurements were used to predict the uniaxial yield stress. The spherical indentation effective stress–strain (${\sigma }_{ind}, {\varepsilon }_{ind}$) curve can be converted to a true stress–strain ($\sigma , \varepsilon$) curve following the work by Patel and Kalidindi [30], Eqs. (12) and (13). This conversion is based on two-dimensional finite element modeling of the indentation test using an isotropic elastic–plastic material with a power law hardening constitutive model. The uniaxial yield stress, ${\sigma }_{ys}$, is half the indentation yield stress, ${\sigma }_{ind,ys}$, which is determined with a 0.2% strain offset on the effective stress–strain curve. The term ${E}_{ind}={E}_{s}/(1-{v}_{s}^{2})$ is just the sample portion of the effective modulus. The Berkovich hardness was used to estimate the uniaxial yield stress using Eq. (15), which is empirically determined from Vickers hardness and tensile data of various reactor pressure vessel steels [31, 32]. The difference in contact area definitions (after unload vs. at maximum load) between Vickers and Berkovich indentation is already accounted for in Eq. (15).

12 $$\sigma =\frac{{\sigma }_{ind}}{2.2}$$

13 $$\varepsilon =\frac{{\sigma }_{ind}}{2.0\left(1-{v}_{s}^{2}\right){E}_{ind}}+\frac{{\varepsilon }_{ind}-\frac{{\sigma }_{ind}}{{E}_{ind}}}{1.3}$$

14 $${\sigma }_{ys}=\frac{{\sigma }_{ind,ys}}{2.0}$$

15 $${\sigma }_{ys}=266.5H-114$$

## Results

### Indentation data

**Figure 2:**
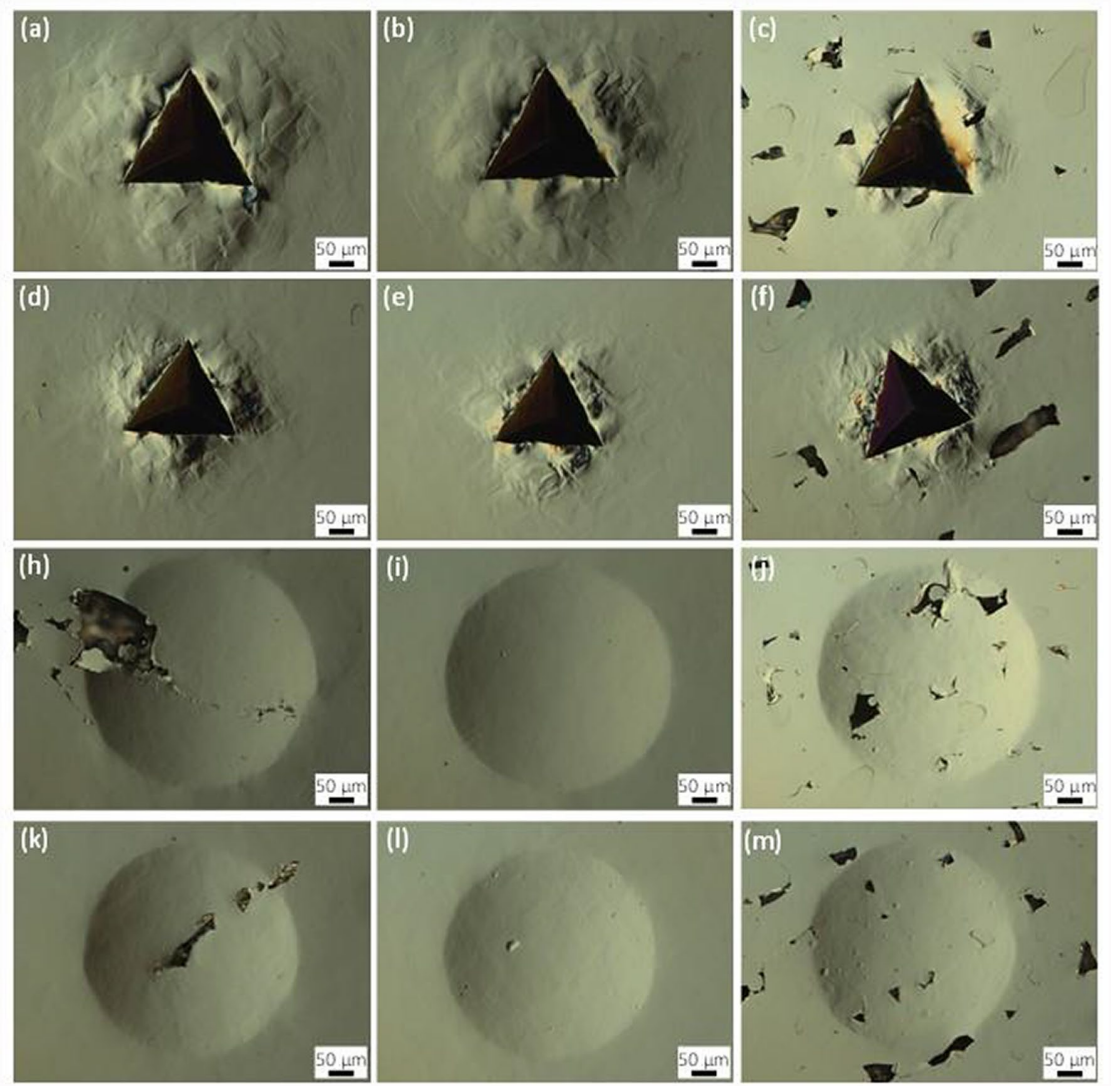
Residual indents for indentation along the Z-direction: Berkovich (**a**-**f**) and spherical (**h**-**m**). The hatch spacing is the same in each column: left, middle, right columns are 50 μm, 80 μm, and 150 μm, respectively. The heat treatment is the same in each row: top to bottom rows are SA, SR, SA, and SR, respectively. The scale bar is 50 μm. Images are circular differential interference contrast (C-DIC) optical micrographs.

**Figure 3:**
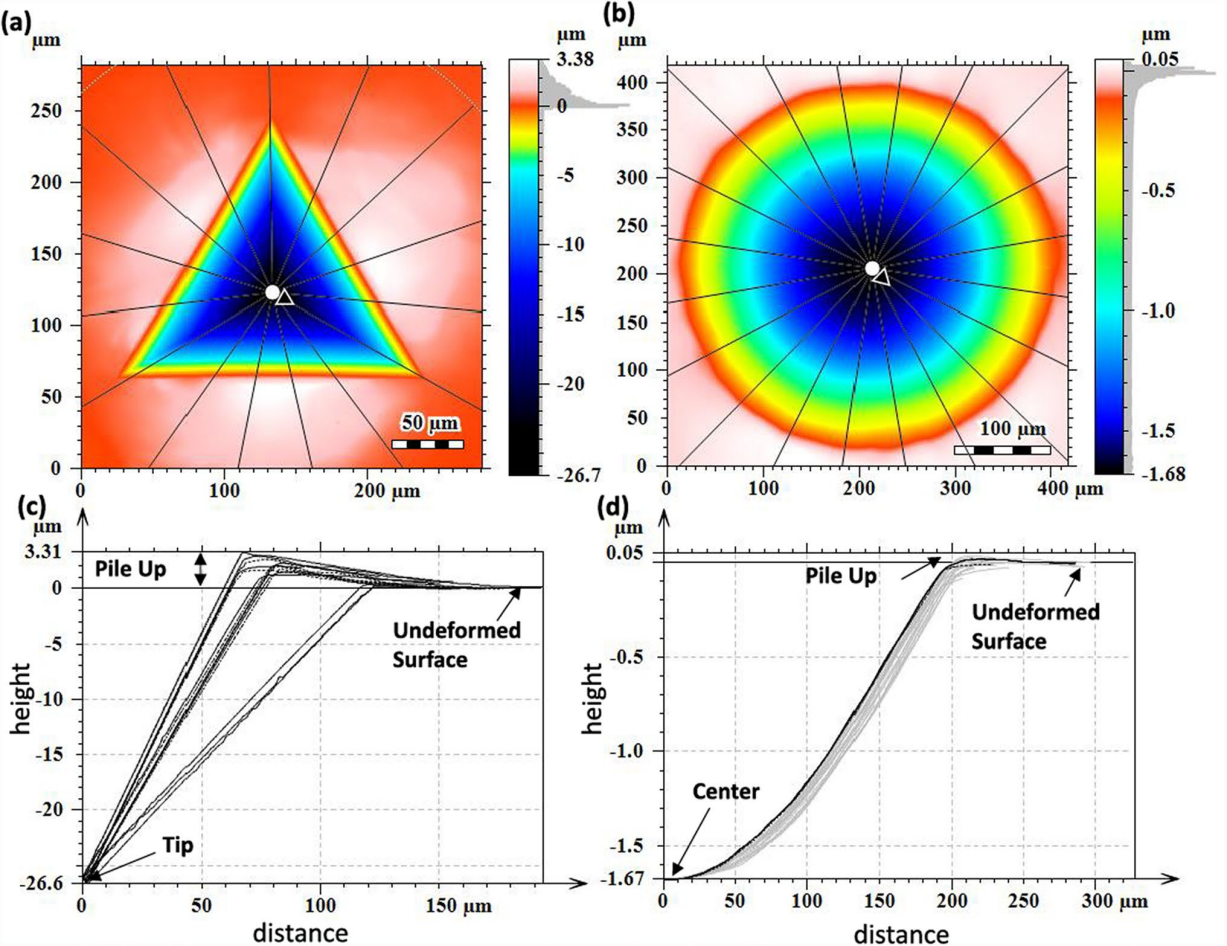
CSI areal height maps of (**a**) Berkovich and (**b**) spherical indents along the X/Y-direction on the SR, 80 μm sample. Line profiles marked on the areal heigh maps are given in (**c**) and (**d**) for the Berkovich and spherical residual indents, respectively. The height is relative to the undeformed surface and the distance is relative to the center of the indent.

Optical images of residual indents are shown in Fig. 2. These give a sense of the scale of the material probed relative to the porosity. It should be noted that this is the scale of material probed at the end of the test, and the material probed is less than this during most of the test. Additionally, these images only show what is on the surface such that porosity may or may not be present below the surface in a way that affects the test result. The far-left column in Fig. 2 contains micrographs for the 50 μm hatch spacing. Many indents appear to land in fully dense regions; however, the indents occasionally interact with large, irregular pores associated with the unstable melting parameters used to print the material (e.g., Fig. 2 h,k). The middle column in Fig. 2 contains micrographs for fully dense material with a hatch spacing of 80 μm. The far-right column contains micrographs for 150 μm hatch spacing with lack-of-fusion porosity. In this case indents almost always cover some porosity defects. The SA material has larger grains and lower strength compared to the SR material, which results in slightly larger indents for the SA material compared to SR: comparing rows 1 and 3 to rows 2 and 4 in Fig. 2, respectively. The difference in grain structure can also be made out in the deformation surrounding the indents, particularly for the Berkovich indents. These examples are for indentation in the Z-direction. CSI areal height maps of a Berkovich and spherical indent on the SR material produced with 80 μm hatch spacing for indentation in the X/Y-direction are shown in Fig. 3. While the cross-sectional area of the residual indents is similar (spherical being slightly larger), the extent of deformation is significantly different. The Berkovich indent leaves a much deeper indent on the order of 25 μm with pile-up surrounding the indent on the order of 3 μm. In contrast, the spherical indent is shallower on the order of 1.5 μm with pile-up less than 0.05 μm. In both cases, the residual indents are not severely misshapen, which indicates typical contact conditions between the indenter and sample.

**Figure 4:**
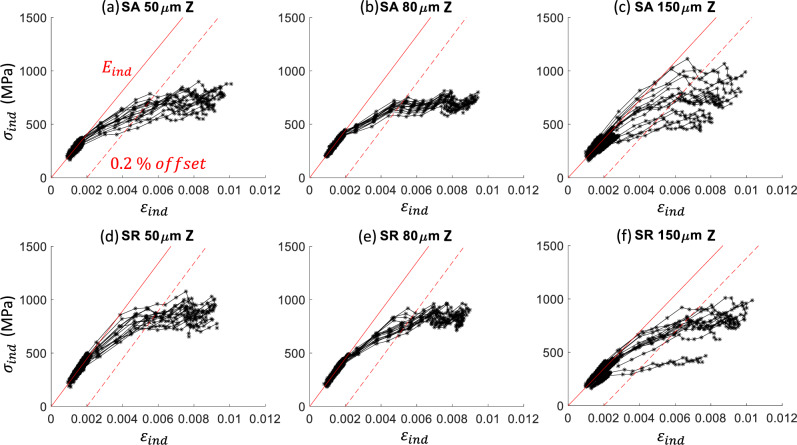
Spherical indentation effective stress–strain (${\sigma }_{ind}, {\varepsilon }_{ind}$) curves for indentation along the Z-direction for the three different hatch spacings (50 μm, 80 μm, and 150 μm corresponding to $\cong$ 1%, < 0.1%, and $\cong$ 4% porosity, respectively) and two heat treatments (SA and SR). The red solid line is the average indentation modulus line (${E}_{ind}$), and the dashed red line is the 0.2% strain offset. These average lines are drawn for illustration; each test was analyzed individually. Each plot has 15 to 16 tests.

**Figure 5:**
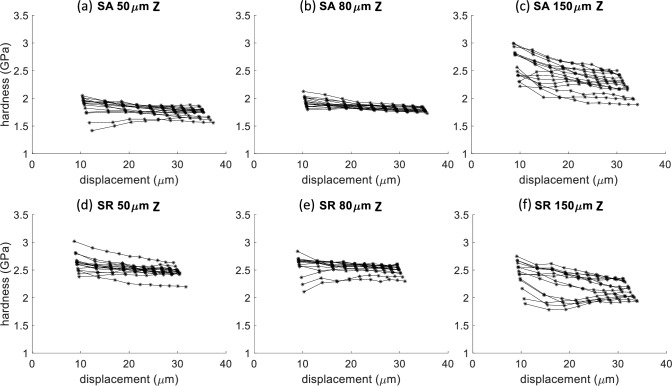
Hardness versus displacement for Berkovich indentation along the Z-direction for the three different hatch spacings (50 μm, 80 μm, and 150 μm corresponding to $\cong$ 1%, < 0.1%, and $\cong$ 4% porosity, respectively) and two heat treatments (SA and SR). Each plot has 14 to 16 tests.

The spherical indents and analysis produce indentation stress–strain (ISS) curves with an initial linear-elastic response followed by a transition to elastic–plastic deformation. The analysis procedure uses a linear regression ($P$ vs. ${h}^{3/2}$) in applying Hertz’s equation (Eq. 3) to the initial loading segment, and it typically produces a goodness of fit R^2^ > 0.98. The indentation stress–strain curves for indents in the Z-direction are shown in Fig. 4. The indentation yield stress is defined by a 0.2% strain offset similar to uniaxial tests. The effect of hatch spacing and thus porosity can be seen in the scatter in the ISS curves. The 80 μm hatch spacing samples with minimal porosity defects have the least scatter in the response, the 50 μm hatch spacing samples have an increase in scatter, and the 150 μm hatch spacing samples have the most scatter with the most porosity defects. The SR material has higher stresses than the SA material due to the refined microstructure (i.e., grain size and dislocation cells) in the SR material. The same trends were observed for spherical indents in the X/Y-direction. The Berkovich indents and analysis produce hardness versus displacement curves shown in Fig. 5. Similar to the ISS curve trends, the hardness data shows increased scatter with porosity and higher hardness for the SR material compared to the SA material.

### Comparison to tensile properties

**Figure 6:**
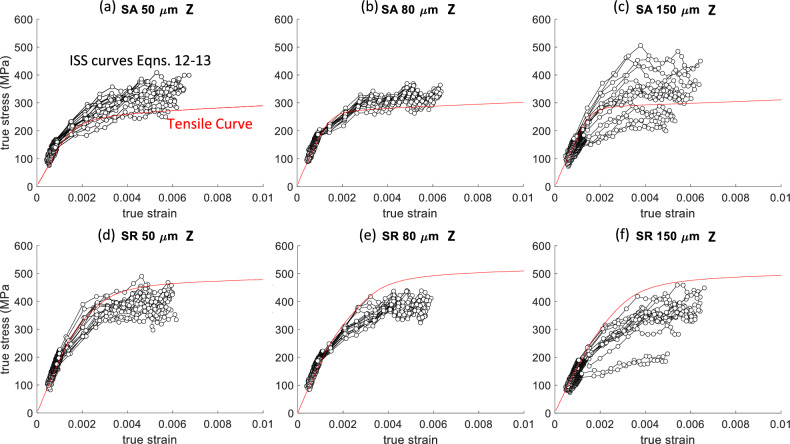
True stress versus true strain comparison of spherical indentation stress–strain (ISS) curves for indents along the Z-direction and a representative tensile curve along the Z-direction for (**a**–**c**) SA condition for all three hatch spacings and (**b**-**d**) SR condition for all three hatch spacings. The hatch spacings 50 μm, 80 μm, and 150 μm correspond to $\cong$ 1%, < 0.1%, and $\cong$ 4% porosity, respectively.

**Figure 7:**
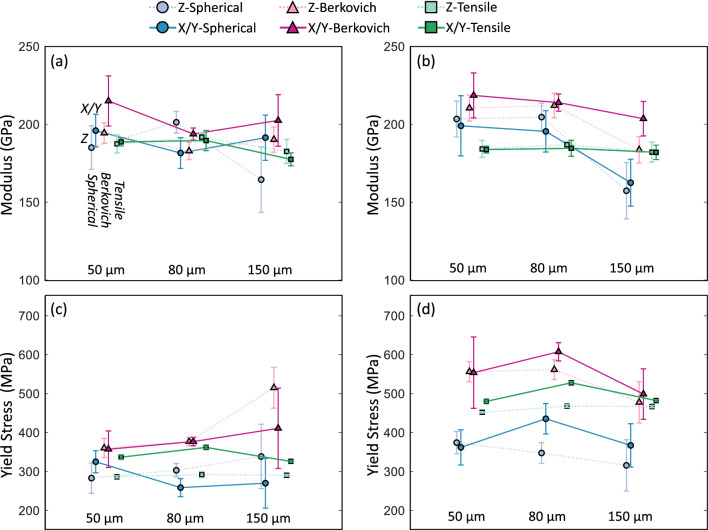
Young’s modulus and yield stress comparisons from spherical ISS, Berkovich hardness, and tensile testing. (**a**, **c**) SA condition (**b**, **d**) SR condition. Data points are the average ± one standard deviation. The hatch spacings 50 μm, 80 μm, and 150 μm correspond to $\cong$ 1%, < 0.1%, and $\cong$ 4% porosity, respectively.

The spherical ISS curves from Fig. 4 were scaled to a true stress–strain curve following Eqs. (12) and (13), and these are referred to as effective stress–strain curves. These are compared against a single representative tensile true stress–strain curve in Fig. 6. This visual comparison shows agreement between the effective stress–strain curves and tensile curves in an average sense for the SA condition across all three hatch spacings. The scaling underpredicts the tensile stress–strain curve for the SR condition. It is not readily apparent why this occurs. Similar results were observed for tests along the X/Y-direction. These are not shown here for brevity; however, the Young’s modulus and yield stress estimates for both test directions are included in the next Fig. 7.

The indentation yield stress and hardness were converted to a uniaxial yield stress using Eqs. (14) and (15). The sample modulus from the indentation analyses does not require a conversion and represents an isotropic elastic modulus. The modulus and yield stress estimates from spherical indentation and Berkovich indentation are compared against the tensile measurements in Fig. 7. The SA and SR conditions are on separate plots (Fig. 7 a,c and Fig. 7b,d, respectively), and the averages for Z and X/Y results are given for each hatch spacing. The modulus results generally agree between the three different measurement methods. For example, the average ± standard deviation percent difference between the indentation and tensile test modulus is 9% ± 4% for Berkovich indents and 1% ± 9% for spherical indents. The yield stress estimates from indentation measurements have a higher percent difference. The average ± standard deviation percent difference is 18% ± 19% for Berkovich indents and -7% ± 17% for spherical indents. Overall, the yield stress from Berkovich indents is at or over the tensile yield stress, and the yield stress from spherical indents is at or below the tensile yield stress.

## Discussion

**Figure 8:**
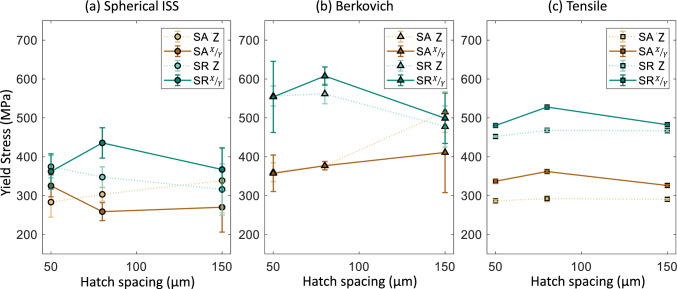
Yield stress estimates and measurements from (**a**) spherical indents, (**b**) Berkovich, and (**c**) tensile tests emphasizing the difference between SA and SR conditions. Data points are the average ± one standard deviation. The error bars on (**c**) are approximately the same size as the markers.

The estimated yield stress from indentation tests captures some of the rankings and trends with specimen orientation and porosity but not all. This is most clearly seen in Fig. 8 where results for spherical, Berkovich, and tensile are on separate plots comparing the SA condition and the SR condition. The heat treatment condition had the greatest influence on the tensile yield stress compared to hatch spacing (porosity). Both spherical and Berkovich yield stress measurements indicate a lower yield stress for the solution annealed (SA) condition compared to the stress-relieved (SR) condition. The difference between the SA and SR is the least for the spherical indent yield stress estimates. Looking at specific cases, when there is a high amount of porosity such as Berkovich 150 μm and spherical 150 μm, the averages converge to similar values for SA and SR conditions. In this case, the large amount of porosity in the indentation zone may dictate the response with less influence from the microstructure differences between the SA and SR solid material. This is essentially a length scale effect that could be addressed with a larger spherical tip; however, to probe a volume comparable to the tensile bar cross section with individual indents defeats the purpose of a local property measurement.

The tensile tests show a mild but statistically significant trend with porosity. The average yield stress reduced for changes in hatch spacing that led to an increase in porosity for all but one case. The horizontally loaded specimens with a hatch spacing of 150 μm have the same yield stress as the 80 μm fully dense specimens. The average yield stress from indentation measurements does not show a clear trend with porosity. This is contrary to Refs. [20–22] that observed a decreasing Vickers hardness with increasing porosity; however, they tested a larger number of samples with different amounts of porosity to reveal a trend. Reiff-Musgrove et al. [33] used a spherical indentation test method called profilometry-based indentation plastometry (PIP) [34] to estimate the tensile properties of metal binder jet additively manufactured 17–4 PH stainless steel. This method uses a spherical tip with a typical radius of 1 mm to much higher strains than what was reached in this study. The higher penetration depths (or strains) are necessary to create a sufficient residual indent for a profile measurement. An iterative finite element method (FEM) analysis is used to match the residual indent profile and extract uniaxial tensile properties. Reiff-Musgrove et al. [33] found that for samples with 1% to 2% porosity, PIP estimates for yield stress predicted yield stress lower than what was measured via tensile tests. One thing that is clear from the spherical indents in this work is that the scatter goes up significantly in the presence of porosity (Fig. 6). Even the Berkovich tests show some increase in scatter (Fig. 5). Again, this can be considered a length scale effect since the bulk tensile tests do not show this level of scatter.

The last trend to discuss is the X/Y versus Z test direction or the anisotropy in yield stress. Mechanical anisotropy is common in many engineering alloys, and alloys produced by L-PBF are no different. Kim et al. [23] report a moderate crystallographic texture of < 110 > along the build direction (Z) and < 111 > along the X- and Y-directions determined from EBSD and X-ray diffraction (XRD). This texture agrees with reports by Charmi et al. [35] who concluded that crystallographic texture was the main reason for anisotropic yield stress in L-PBF 316L stainless steel, which was supported by crystal plasticity simulations. The yield stress anisotropy can be reduced through homogenization and annealing as demonstrated by Ronneberg et al. [18]. However, Kim et al. [23] reported that crystallographic texture remains in the material after the solution annealing (SA) heat treatment used in this study, and therefore anisotropic yield stress remained. Here we note that the stress field under the indenter is not uniform, so it is not expected to be as sensitive to yield stress anisotropy as a tensile test, which applies a uniform stress along a single direction. Therefore, it is very possible that tensile tests reveal some anisotropy while indentation tests do not. Indentation of individual grains are a benchmark for how much anisotropy can be expected. Tekumalla et al. [36] characterized L-PBF 316L stainless steel with Berkvoich nanoindentation inside individual grains reporting a hardness range from 2.6 GPa along the < 100 > direction to 3.0 GPa along the < 111 > direction. Indents along < 110 > direction had average hardness average only slightly lower than the < 111 > direction. Additionally, they found that the cellular structure inside grains was a factor causing anisotropic hardness based on crystal plasticity simulations of indents. Uddin et al. [37] found similar results for L-PBF 316 stainless steel; however, they report hardness values for (110) grains to be more like the lower hardness values of (100) grains rather than the higher hardness values of (111) grains. In traditionally manufactured 316L stainless steel, Stinville et al. [38] conducted instrumented indentation of individual grains using a Vickers tip geometry with 100 mN maximum applied force. They found a linear trend of hardness versus an anisotropic (orientation) factor with the lowest hardness of approximately 1.8 GPa for (100) grains, the highest hardness of approximately 2.2 GPa for (111) grains, and the hardness of (110) grains in-between. The yield stress estimates from indentation results in this study do not show a dependence on the test direction meaning they do not capture the anisotropic tensile yield stress. Recall, the material in this study has a moderate crystallographic texture of (110) along Z-direction and (111) along X/Y-direction. Therefore, we estimate the maximum potential hardness anisotropy to be approximately 0.2 GPa based on single grain results in literature for (110) versus (111). Since the crystal texture is only moderate, and the indent probes a polycrystalline volume (many grains under the indenter compared to a single grain), we expect the plastic anisotropy to be less than single grain tests. Therefore, it is reasonable that the yield stress estimates from indentation do not follow the tensile test trend with test direction. Additionally, the conversions used in this study for estimating yield stress from indentation measurements do not account for mechanical anisotropy. A more sophistical model is required if significant anisotropy is present in the indentation response.

## Conclusions

High-load (50–150 N) instrumented indentation with Berkovich and spherical tip geometries was used to estimate the modulus and yield stress of L-PBF 316L stainless steel with varying types of porosity created by decreasing and increasing hatch spacing. Indentation testing was carried out along the Z and X/Y-directions and compared against uniaxial tensile test results for two different heat treatment conditions (stress-relieved and solution annealed). The largest change in yield stress was observed between the two heat treatment conditions, and the indentation measurements pick up on this relative difference. The average yield stress estimated from spherical indentation was at or below the tensile yield stress and estimates from Berkovich indentation were at or above the tensile yield stress. For the highest porosity level, the difference between heat treatments was not distinguishable with indentation measurements. The tensile yield stress was moderately reduced for conditions with porosities of 1% and 4%. The average indentation results did not show a clear trend with porosity; however, the scatter from test to test increased significantly with the presence of porosity. The largest scatter was seen for spherical indentation stress–strain curves with the largest porosity. This is a length scale effect, where the indentation test probes fully dense and highly porous regions in the interaction volume depending on the test location. The porosity had the biggest effect on the elongation to failure; however, the indentation methods used in this study do not estimate this property. The indentation measurement methods also do not account for anisotropy in yield stress and are less sensitive to anisotropic yield stress due to the non-uniform stress under the indenter. This resulted in no trend with test direction for indentation measurements. The modulus results from indentation measurements generally agreed with the tensile test measurements. The indentation methods can provide a means for low cost, rapid feedback in L-PBF materials that may contain porosity defects; however, further work is needed to either improve or quantify the uncertainty in yield stress predictions. FEM of indentation with porosity defects will also provide much needed insight and improvements in the methodology.

## Data Availability

Data are available upon reasonable request.

## Footnotes

1 Certain equipment, instruments, software, or materials are identified in this paper in order to specify the experimental procedure adequately. Such identification is not intended to imply recommendation or endorsement of any product or service by NIST, nor is it intended to imply that the materials or equipment identified are necessarily the best available for the purpose.
